# Biological and genomic analysis of a symbiotic nitrogen fixation defective mutant in *Medicago truncatula*

**DOI:** 10.3389/fpls.2023.1209664

**Published:** 2023-06-28

**Authors:** Yitong Shen, Yelin Ma, Dengyao Li, Mingming Kang, Yue Pei, Rui Zhang, Weiyu Tao, Shenxi Huang, Wenjie Song, Yuecheng Li, Wanqi Huang, Duanyang Wang, Yuhui Chen

**Affiliations:** Ministry of Education Key Laboratory of Cell Activities and Stress Adaptations, School of Life Sciences, Lanzhou University, Lanzhou, Gansu, China

**Keywords:** *Medicago truncatula* (barrel medic), symbiotic nitrogen fixation (SNF), nodule development, legume, whole genome sequencing (WGS)

## Abstract

*Medicago truncatula* has been selected as one of the model legume species for gene functional studies. To elucidate the functions of the very large number of genes present in plant genomes, genetic mutant resources are very useful and necessary tools. Fast Neutron (FN) mutagenesis is effective in inducing deletion mutations in genomes of diverse species. Through this method, we have generated a large mutant resource in *M. truncatula*. This mutant resources have been used to screen for different mutant using a forward genetics methods. We have isolated and identified a large amount of symbiotic nitrogen fixation (SNF) deficiency mutants. Here, we describe the detail procedures that are being used to characterize symbiotic mutants in *M. truncatula*. In recent years, whole genome sequencing has been used to speed up and scale up the deletion identification in the mutant. Using this method, we have successfully isolated a SNF defective mutant FN007 and identified that it has a large segment deletion on chromosome 3. The causal deletion in the mutant was confirmed by tail PCR amplication and sequencing. Our results illustrate the utility of whole genome sequencing analysis in the characterization of FN induced deletion mutants for gene discovery and functional studies in the *M. truncatula*. It is expected to improve our understanding of molecular mechanisms underlying symbiotic nitrogen fixation in legume plants to a great extent.

## Introduction

Fast neutron produced from nuclear fission reactors or particle accelerator spallation sources are a type of high-energy ionizing irradiation effective in generating mutations in genomes of diverse organisms (Men et al., 2002). The nature of mutations induced by FN mutagenesis includes deletions of various sizes and other mutation types such as inversion, translocation and duplication (Bruggemann et al., 1998). Deletions cause gene loss or disruption and are especially valuable in functional studies of individual and tandemly-duplicated genes. Several large FN mutant populations have been developed in different plant species including *Arabidopsis thaliana*, *Oryza sativa L.*, *Glycine max*, *Lotus japonicus*, *barley* and *M. truncatula* (Li et al., 2002; Alonso et al., 2003; Mitra et al., 2004; Rostoks et al., 2006; Wang et al., 2006; Hoffmann et al., 2007; Bolon et al., 2011).

*M. truncatula* is also an ideal model species for studying legume nodule development and symbiotic nitrogen fixation (SNF) (Cook, 1999). The symbiotic interaction between *M. truncatula* and rhizobia forms indeterminate nodules, which can be divided into five main zones: meristem, infection, intermediate transition, nitrogen fixation, and senescence. The apical meristem zone is mainly responsible for cell division and new cell supply for nodule development. The infection zone is the entry point for rhizobia into nodule cells through infection threads, and the transition zone is where rhizobia enter nodule cells and form symbiont. After entering the nitrogen fixation zone, bacteroids play a role in converting atmospheric nitrogen into ammonia, which can be used directly by plants. Finally, the senescence zone is where symbiont and bacteroids degrade and lose their nitrogen-fixing activity. Clearly, the symbiotic nitrogen fixation process is very delicate and complex, and depends on the mutual recognition between plant and rhizobia at the molecular level for signaling and metabolite exchange. The symbiotic relationship will cease if there are problems in any of the above areas. Loss of symbiotic nitrogen fixation in legumes can lead to the formation of different types of mutants, which are characterized as the three main categories: (1) non-nodulating mutants (Nod-), (2) nodulating but not nitrogen-fixing mutants (Nod+/Fix-), and (3) mutants with excessive nodulation (Nod+++) (Oldroyd, 2013).

Years of mutant analyses have led to identification of genes and molecular mechanisms involved in nodule initiation and development, including mutual recognition of rhizobia and root hairs. Mutants from the early recognition process mainly affect nodule formation, but not the nitrogen fixation (Mergaert et al., 2020). However, there are relatively less studies on genes and their mechanisms involved in rhizobia release to infected nodule cells and nodule senescence. Therefore, studies on the late development of legume-rhizobia symbiotic nitrogen fixation have become a major direction in legume research (Roy et al., 2020).

The establishment of an FN mutant library in *M. truncatula* provides opportunities for studying gene functions, and a large number of mutants with symbiotic nitrogen fixation deficiency phenotypes provide a reliable guarantee for identifying gene functions and studying molecular regulatory mechanisms (Chen and Chen, 2018). However, molecular cloning of genes from FN mutants traditionally relies on the map-based cloning approach, which is a time-consuming technique that limits the amount of mutants to be analyzed at the molecular level. Several complementary approaches, such as transcript-based deletion detection and DNA copy number variation detection using a whole genome tiling array comparative genomic hybridization (CGH) platform, have been developed to facilitate the characterization of deletion mutants in diverse organisms including animals and plants (Geschwind et al., 1998; Infante et al., 2003; Gong et al., 2004; Werner et al., 2005; Bejjani and Shaffer, 2006; Lakshmi et al., 2006; Emerson et al., 2008; Perry et al., 2008).

The array-based CGH (aCGH) method is a powerful tool for the analysis of copy number variation, polymorphisms, and structural variations in the genome (Bolon et al., 2011; Haun et al., 2011; Horvath et al., 2015; Kim et al., 2015). However, low accuracy and low sensitivity for small deletions by the CGH approach are apparent. It has a higher probe density in exons than in introns and 5’ and 3’ UTRs, which limits the detection of sequence variations to only covered regions (Chen et al., 2017).

Recently, the whole genome sequencing (WGS) platform has delivered comprehensive genetic analyses in humans, plants and animals (Sun et al., 2018). Paired-end whole genome sequencing involves sequencing both ends of a DNA fragment, which increases the likelihood of alignment to the reference genome and facilitates detection of genomic rearrangements, repetitive sequences, and gene fusions. Unlike focused approaches such as exome or targeted resequencing, which analyze a limited portion of the genome, whole genome sequencing conveys a comprehensive view of the entire genome. It is ideal for discovery applications, such as identifying genome deletions and duplications. Whole-genome sequencing can detect single nucleotide variants, insertions, deletions, copy number changes, and large structural variants. The application of WGS platforms to FN mutant populations can identify and locate genomic changes at the whole genome level (Du et al., 2021).

Here, we isolated and characterized of the *M. truncatula* FN007 mutant from fast neutron deletion mutant library. The FN007 mutant showed defective in biological nitrogen fixation and nodule development associated with early senescence phenotype. Detailed phenotypic analysis was carried out in the FN007 mutant and would provide new insight of symbiotic nitrogen fixation processes in leguminous plants. Furthermore, the FN007 mutant was analyzed by WGS platform to detect genomic alterations. Our results confirmed that the WGS platform was efficient in mapping genome deletions and would greatly facilitate characterization of a large number of FN mutants for gene functional studies in *M. truncatula*. Using this mutant screening and identification method should improve our understanding of molecular mechanisms underlying symbiotic nitrogen fixation.

## Materials and methods

### Plant materials and growth condition

*M. truncatula* seeds were scarified with concentrated sulfuric acid for 5 to 8 min, rinsed with deionized water for five times, surface sterilized with 5% sodium hypochlorite solution and rinsed with autoclaved deionized water for several times. After vernalization at 4°C for 3 days, seedlings were transplanted to pots and grown in greenhouse with 16 hr/8 hr light/dark cycle and 150 E/m2/sec light intensity. Perlite and sand (volume ratio of 3:1) were used as the planting medium. The perlite and sand were separately rinsed with deionized water, and then mixed in proportion for use. The germinated seeds were transferred to a 72-cell tray (530 mm × 270 mm, with a single well volume of 40 mL), watered with 1 L of 0.125 mM KNO_3_ (low-nitrogen nutrient) solution. Rhizobia were inoculated after one week of growth in the greenhouse.

### Inoculation with the rhizobial: *Sinorhizobium medicae* ABS7 strain and *Sinorhizobium meliloti* Sm2011-GFP strain

The rhizobia strains were removed from a -80°C refrigerator. Two mL of the rhizobia were inoculated into 50 mL of TY liquid medium containing tetracycline and cultured at 28°C for 2 days. Then, the bacterial cultures were centrifuged at 4,000 rpm for fifteen min in a 50 mL centrifuge tube, re-suspended and diluted to OD600≈0.05 with the low nitrogen nutrient solution. Five mL of the above bacterial suspension were inoculated to each plant.

### Microscopic observations

Three to five plants inoculated with *S. medicae* ABS7 strain carrying the *hemA::LacZ* reporter were harvested at 6 dpi, 8 dpi, 10 dpi, 13 dpi and 16 dpi (days post inoculation). Roots were washed with deionized water. The root nodules were fixed as previously described (Xi et al., 2013). For GFP studies, nodules from plants inoculated with the *S. meliloti* Sm2011 strain harboring *hemA::mGFP* reporter gene were washed with deionized water, embedded in 6% agarose, and sectioned with Vibratome™ 1000 Plus (Leica Biosystems, Germany). Nodule sections (50-70 μm) were mounted on glass slides and imaged using MVX10+DP74 microscope (Olympus).

### Live/dead staining of nodule cells and potassium iodide staining

Plants inoculated with *S. medicae* ABS7 strain were used for 50-70 μm nodule section preparation. 5 μM SYTO9 and 30 μM PI were added to 50 mM Tris (pH7.0) buffer to make a staining solution. Nodule sections were stained for 20 min at room temperature. Sections were placed on slides covered with coverslips and observed with a confocal microscope [Nikon Confocal laser scanning microscope (A1R+ Ti2-E)].

Five-micrometer semi-thin sections were placed on glass slides and stained with 1% potassium iodide phosphate buffer in dark condition for 10 min. Then, the image was observed with microscope [Axio Imager.Z2 (ZEISS)].

### Semithin section of root nodules and toluidine blue staining

The nodules were placed in the fixing solution, vacuumed for 10 min, and fixed overnight at 4°C. The fixed nodules were dehydrated in 70%, 85% and 95% ethanol series for 30 min each, and in 100% ethanol for 2 h. Finally, 0.1% eosin Y3 was added to 100% ethanol to dye for half an hour. After the nodules turned red, samples were replaced in a mixture of 100% ethanol and base liquid Technovit 7100 resin (v/v=1:1) for 2 h. The nodule samples were transferred to the infiltration solution overnight (infiltration solution: 1 g hardener I to 100 mL base liquid Technovit 7100 resin. Allow approximately 5 min for complete dissolution). The nodule samples were embedded after the permeation treatment. The temperature was set to 40°C and the embedding mould was dried beforehand. The infiltrate and hardener II (v/v=15:1) were mixed gently, and 200 μL of the mixture was added to each well of the embedding mould. The nodule materials were carefully picked up with forceps and placed on the tip of the embedding mould, with materials placed vertically to facilitate sectioning. The samples were arranged neatly and then polymerized overnight in a 40°C incubator. The Leica RM2265 rotary microtome was used to cut sections with a thickness of 3-5 μm. The semithin sections were placed on glass slides and stained with 1% toluidine blue for 5 min. Finally, the stained sections were observed and imaged with microscope [Axio Imager.Z2 (ZEISS)].

### Whole genome sequencing analysis

The whole genome sequencing (WGS) analysis was performed by Shanghai Oebiotech Co. The compound leaf samples were collected from wild type (WT) A17 and FN007 mutant, respectively. The genomic DNA was extracted using the DNeasy plant mini kit (Qiagen Shanghai, China), following the supplier’s instructions. The library was constructed after the DNA quality was passing the bioanalyzer test. The DNA samples were randomly sheared into 350 bp-500 bp fragments, and the TruSeq DNA LT Sample Prep Kit was used for library construction. The DNA fragments were treated with the following steps to complete library construction: end repair, addition of ployA tails, addition of sequencing connectors, purification, and PCR amplification. After library construction and qualification, pair-end sequencing was performed on the sequencer (HiSeq X Ten sequencer).

The raw sequencing data were processed in two stages. Sequencing data quality was mainly examined through the sequencing error rate, data volume, comparison rate, coverage and other statistics to assess whether the sequenced library meet the standard for subsequent analysis. Then, the high-quality sequences were compared to the reference genome to detect the variation information in the samples. SNP (single nucleotide polymorphisms) and InDel (insertion or deletion) detection was performed using the GVCF mode of the Haplotypecaller module of the GATK (Version: 4.1.0.0) software, based on the results of the sample comparison with the reference genome. To reduce the error rate of SNP and InDel assays, a criterion of QD more than 2.0 was chosen for filtering, and only mutant loci that met this criterion were retained. QD is the ratio of the quality of variation divided by the depth of coverage, which is actually the quality of variation per unit depth. The SNP and InDel results were annotated using SnpEff (Version: 4.4) software. Finally, the genomic variation information detected was visualized using Circos plots.

To eliminate the effect of sequencing errors on the results, the original sequencing data were cleaned using the pre-processing software Fastp. Clean reads were compared to reference genome using BWA, and the comparison results were converted to SAMtools, and de-redundant using Picard (http://broadinstitute.github.io/picard/), and the results were analyzed using the Qualimap software (Li and Durbin, 2009; Li et al., 2009; Chen et al., 2018). *M. truncatula* reference genome can download at the website: https://medicago.toulouse.inra.fr/MtrunA17r5.0-ANR/(MtrunA17r5.0).

### TAIL PCR

Thermal asymmetric interlaced PCR (TAIL PCR) assay was carried out essentially as previously described (Liu and Chen, 2007). The specific primer P1 adjacent to the proposed deletion border was combined with five short randomly primers (AD1, AD2, AD3, AD5, AD6) with low Tm values and amplified by PCR with thermally asymmetric temperature cycling, using the FN007 mutant genomic DNA as template.

The resulting PCR product was amplified again to obtain a flanking sequence using the nested primer P2 of P1 (**Table S1**) and the five random primers mentioned above. The PCR product was sequenced and compared with the genome sequence of *M. truncatula* A17 to confirm the deletion junctions in FN007 mutant.

## Results

### Phenotypes of FN007 mutant under low nitrogen condition

**Figure 1:**
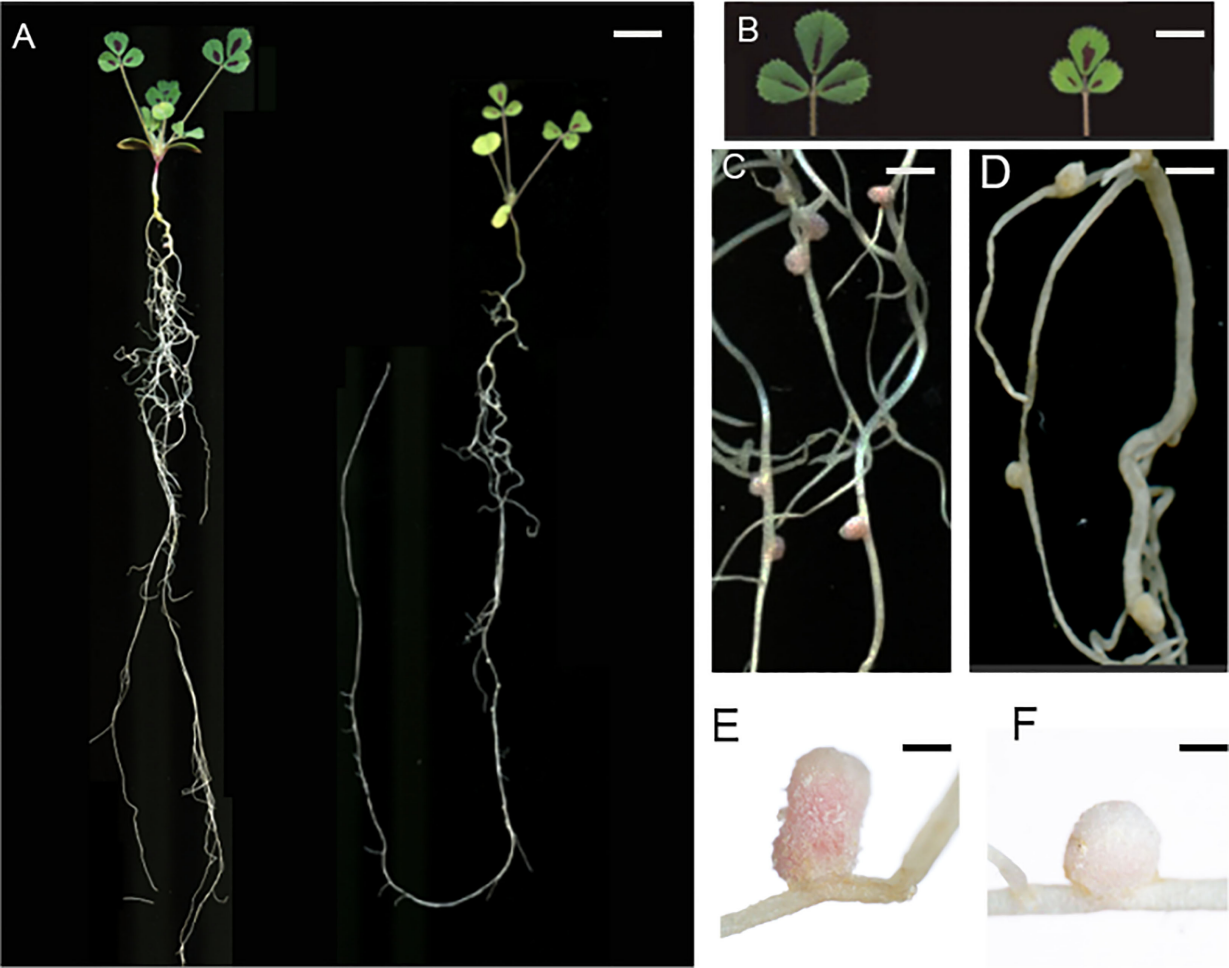
*M. truncatula* FN007 mutant phenotype. **(A)** Growth of FN007 (right) and wild type (WT, left) seedlings 16 days post inoculation (dpi) with *S. medicae* ABS7 strain, showing dwarf phenotype of FN007 mutant. **(B)** leaf phenotype of FN007 mutant (right) and WT (left), yellowish leaves are the sign of nitrogen deficiency phenotype. Nodules developed on **(C)** (WT) and **(D)** (FN007 mutant) were pink and white color, respectively. Close-up view of **(E)**, WT and **(F)**, FN007 mutant nodules. Scale bar: **(A)**, 1 cm; **(B)**, 50 mm; **(C, D)**, 10 mm; **(E, F)**, 1 mm.

*M. truncatula* FN007 mutant was isolated from a Fast Neutron (FN) induced deletion mutant collection in the Jemalong A17 background. Under Nitrogen replete condition, the FN007 mutant is comparable with Wild Type (WT) plants in growth and development processes, including leaf, seed pod, flower and root development (data not shown). But under Nitrogen-limited and symbiotic condition, the FN007 mutant plant develop nitrogen starvation symptoms including stunted growth and yellowish leaves at 16 days post inoculation (dpi) with *S. medicae* ABS7 strain, indicating that these phenotypes were caused by defective symbiotic nitrogen fixation (**Figures 1A, B**). Nodules developed on the FN007 mutant roots were small and spherical in shape, most with white colors (due to degradation of leghemoglobin), whereas in the WT plant, the pink cylindrical nodules were developed (**Figures 1C–F**). Moreover, nodule distribution was concentrated in WT plants but scattered in FN007 mutant and the WT plant had fewer nodule numbers than the FN007 mutant.

### Time-course observation of nodule phenotype

**Figure 2:**
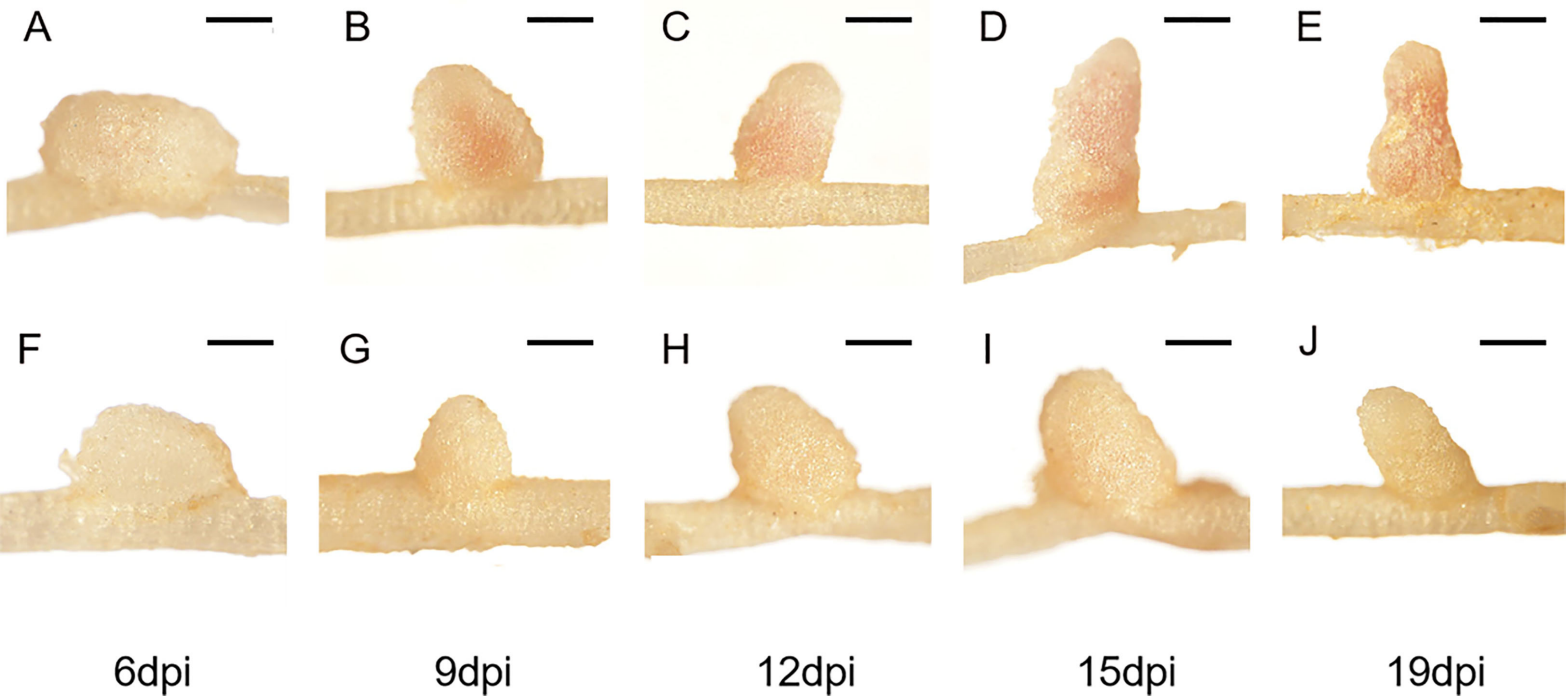
Time-course observations of nodule phenotypes. *M.truncatula* WT and FN007 mutant seedlings were inoculated with *S. medicae* ABS7 strain and inoculated root nodules were observed in a time-course assay. Representative images of **(A)**, WT and **(F)**, FN007 mutant roots 6 dpi; **(B)**, WT and **(G)**, FN007 mutant 9 dpi; **(C)**, WT and **(H)**, FN007 mutant 12 dpi; **(D)**, WT and **(I)**, FN007 mutant 15 dpi; **(E)**, WT and **(J)**, FN007 mutant 19 dpi. These observations suggest that FN007 mutant has an abnormal nodule development. Scale bar: 500 μm.

To further characterize the nodule development processes of the FN007 mutant, time-course experiments were carried out. Under low nitrogen condition (0.125 mM KNO_3_), the growth and development of root nodules in the FN007 mutant and WT plant inoculated with *S. medicae* ABS7 strain was continuously monitored and recorded. Morphological and color differences between FN007 mutant and WT nodules became apparent over time (**Figure 2**). At 6 dpi, WT (**Figure 2A**) and FN007 mutant (**Figure 2F**) nodules appeared to be similar, both white and spherical shape. At 9 dpi, WT nodules (**Figure 2B**) became pale pink color and conical in shape, while FN007 mutant nodules (**Figure 2G**) were still white color and cone-shaped. At 12dpi, WT nodules (**Figure 2C**) were pink and cylindrical shape, but FN007 mutant nodules (**Figure 2H**) gradually increased in size with increasing growth time, and remained white color. At 15 dpi, WT nodules (**Figure 2D**) continued to grow, almost entirely pink, and were already mature and capable of efficient nitrogen fixation. FN007 mutant nodules (**Figure 2I**) grew slowly, with one or two white and rod-like, and the remainder were basically white spheres or cones shape. At 19 dpi, WT nodules (**Figure 2E**) were generally the same as those at 15 dpi, but the number of mature nodules increased, while FN007 mutant nodules (**Figure 2J**) still appeared as white rod shape. Time-course experiments showed that early nodule development (6 dpi) appears to be normal in the FN007 mutant. However, differences began apparent at 6-9 dpi with leghemoglobin accumulation in WT nodules. Even though the FN007 nodules did not show the pink functional leghemoglobin, some nodules were able to elongate and grow into rod-like shape during later development stage, indicating that the nodule meristem still had normal developmental functions.

**Figure 3:**
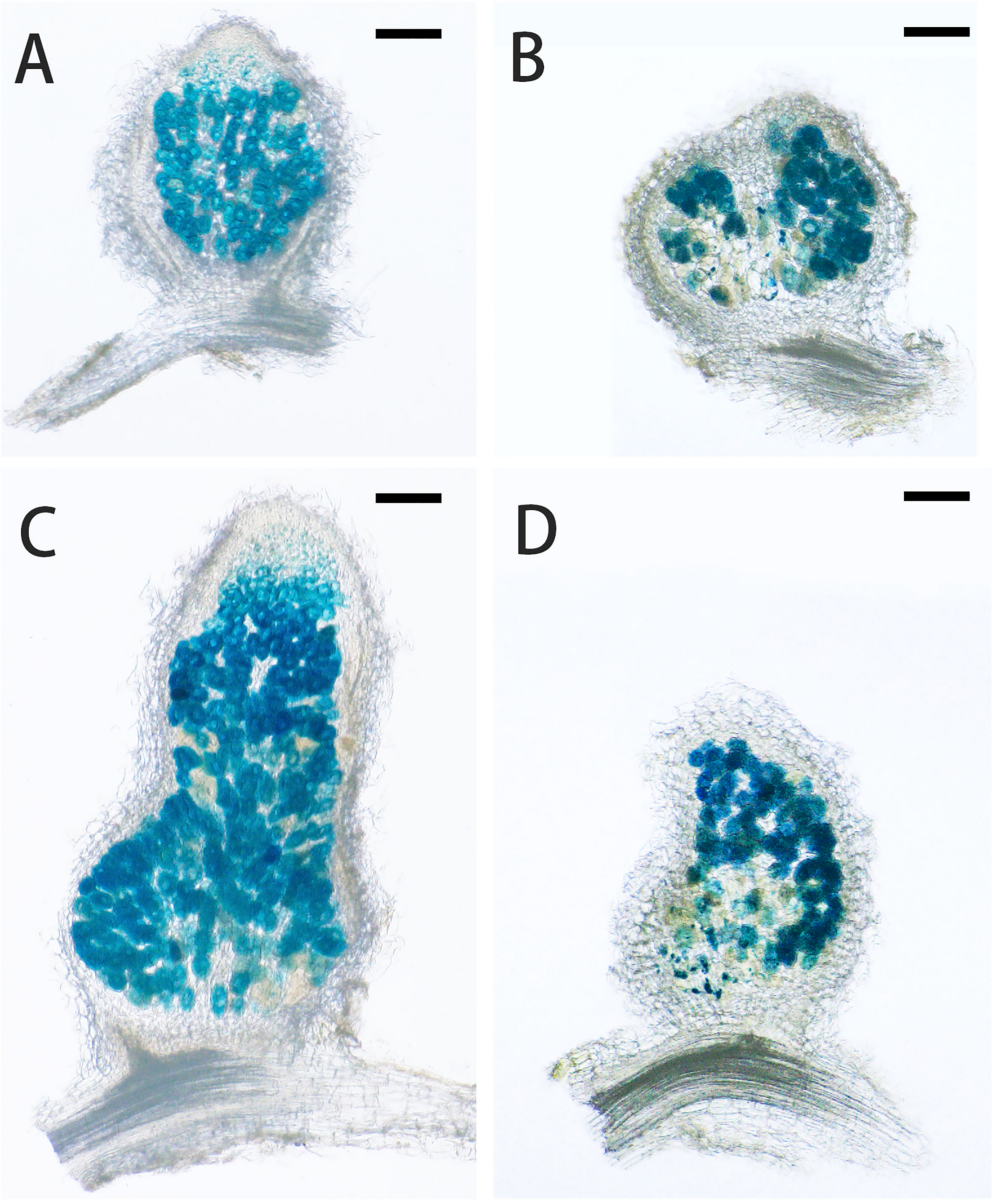
*Lac*Z stained sections observation of nodule development. *M.truncatula* WT and FN007 mutant seedlings were inoculated with *S. medicae* ABS7 strain harboring *hemA::Lac*Z reporter gene. Longitudinal sections of nodules were observed under a MVX10+DP74 microscope and representative images were shown. **(A)**, WT and **(B)**, FN007 mutant nodules 12 dpi; **(C)**, WT and **(D)**, FN007 mutant nodules 15 dpi. Scale bar: 200 μm.

**Figure 4:**
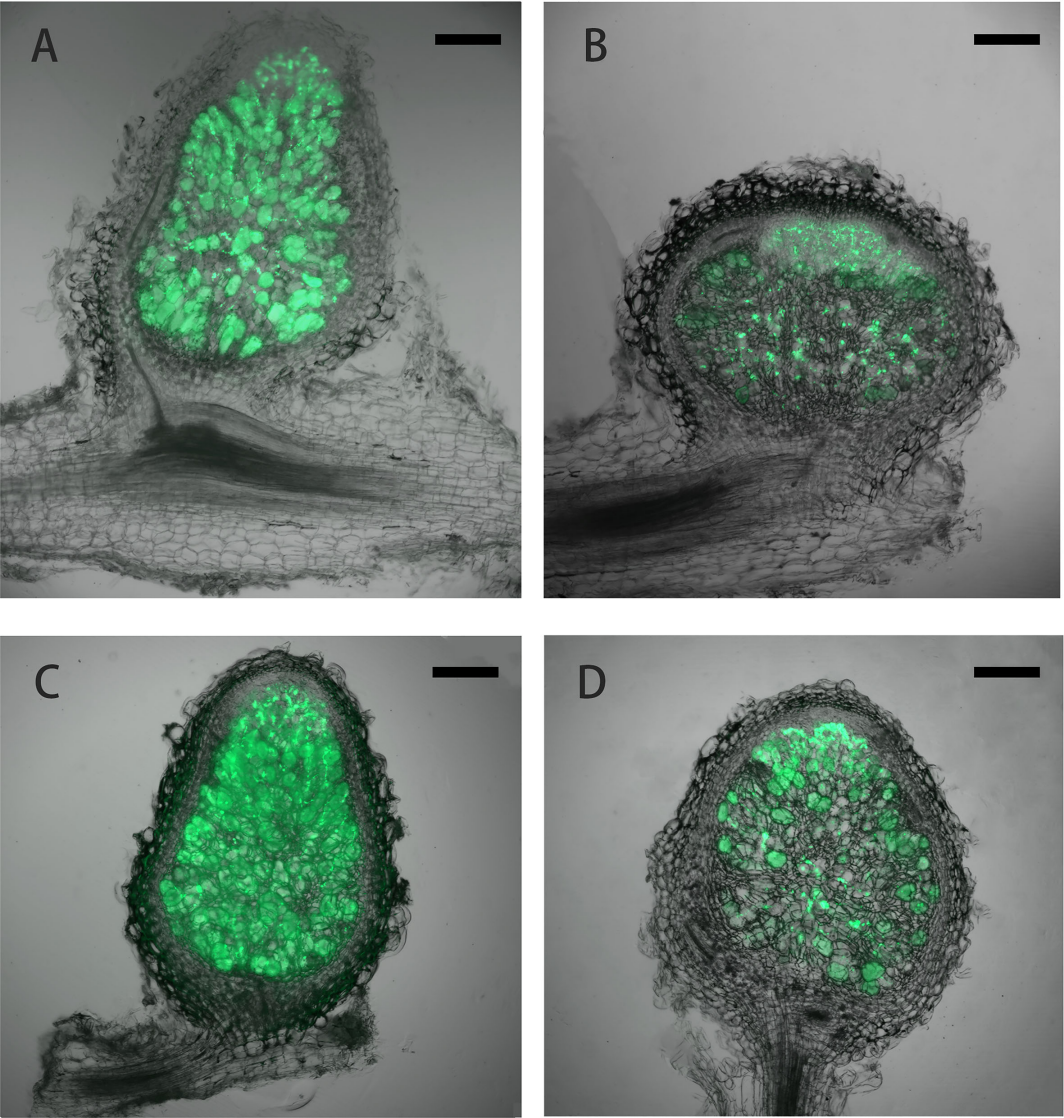
Laser confocal microscopic analysis of nodule development. *M.truncatula* WT and FN007 mutant seedlings were inoculated with *S. meliloti* Sm2011 strain harboring *hemA::mGFP* reporter gene. Longitudinal sections of nodules were observed under a MVX10+DP74 microscope and representative images were shown. **(A)**, WT and **(B)**, FN007 mutant nodules 11 dpi; **(C)**, WT and **(D)**, FN007 mutant nodules 14 dpi. Scale bar: 200 μm.

Under low nitrogen condition (0.125 mM KNO_3_), the FN007 mutant and WT seedlings grown for one week in greenhouse were inoculated with *S. medicae* ABS7 strain containing the *hemA::LacZ* reporter gene. Nodules were harvested at different development stages after inoculation and stained with *LacZ* staining buffer. At 12 dpi, in FN007 mutant nodules (**Figure 3B**), there were none or few rhizobia in nodule cells. For WT plant at the same time (**Figure 3A**), rhizobia filled the entire nodule cells. At 15 dpi, the number of rhizobia-containing nodule cells in FN007 mutant (**Figure 3D**) was decreased, and nodule cell debris was observed, at which point a distinct senescence zone (rather than a nitrogen-fixing zone) had formed, which was significantly different from the WT (**Figure 3C**). We speculated that between 6-7 dpi, the FN007 mutant failed to form a nitrogen-fixing zone and went directly into senescence, forming a region that accounted for an increasing proportion of nodules over time. To confirm this result, another rhizobia strain with GFP reporter gene, *S. meliloti* Sm2011-GFP, was inoculated. At 11 dpi, the fluorescence signal in wild type nodules was very strong, indicating that rhizobia released and filled with nodule cells (**Figure 4A**). However, in FN007 mutant nodules, though the fluorescence intensity of zones I and II was no different from the WT, zone III fluorescence was distributed in a punctiform pattern and no mature symbiont was formed (**Figure 4B**), indicating that rhizobia release into nodule cells during infection was normal, which was consistent with the results of nodule sections inoculated with *S. medicae* ABS7 strain. At 14 dpi, the WT nodules showed characteristic partitioning and a dominant green fluorescent signal in mature symbiotic cells completely filled with bacteroids (**Figure 4C**), whereas in the FN007 mutant nodules an increasingly severe degradation of the bacterial cycle in the nitrogen-fixing zone was observed (**Figure 4D**). This observation suggests the FN007 mutant nodules may undergo early senescence.

### *M. truncatula* FN007 mutant is defective in symbiotic nitrogen fixation

**Figure 5:**
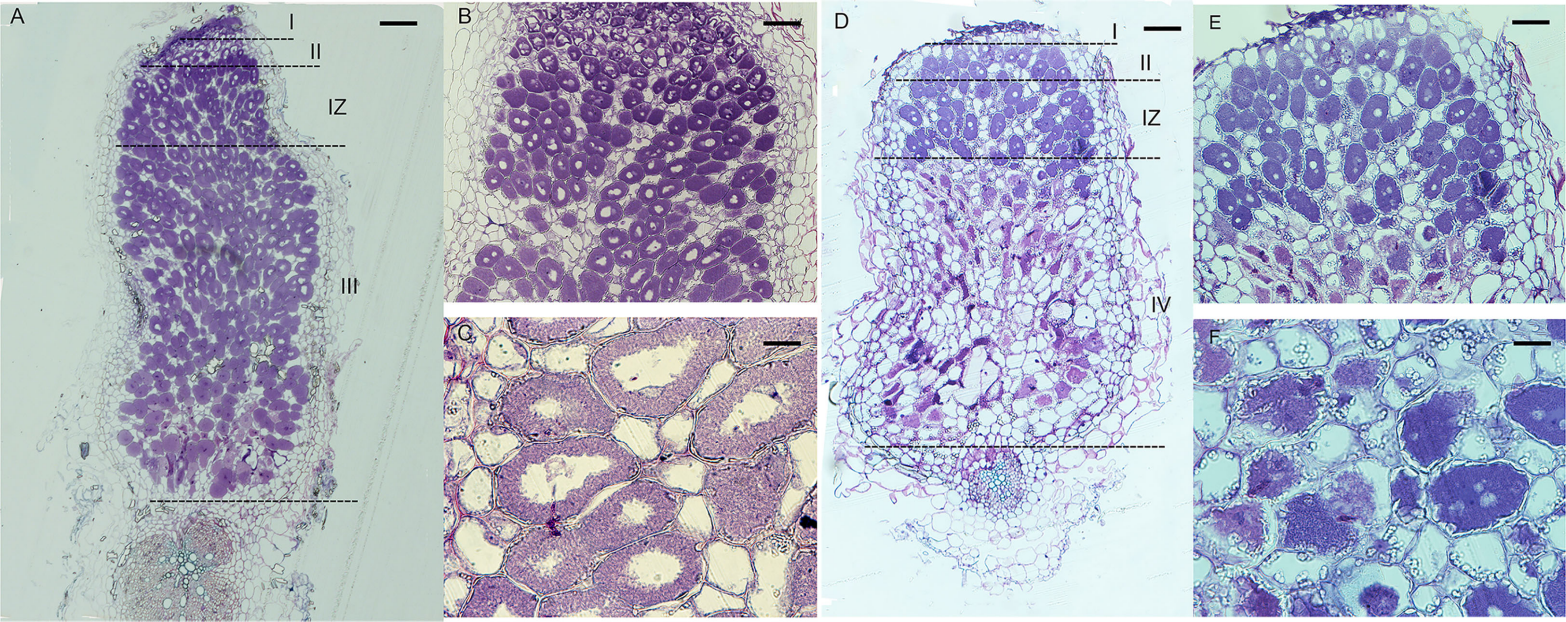
Semithin sections of WT and FN007 mutant nodules. *M. truncatula*, **(A)**, WT and D, FN007 mutant nodules 13 days post inoculation (dpi) were sectioned longitudinally and stained with toluidine blue, showing development of meristem **(I)**, infection (II) and transition zones (IZ), and infected cells in both WT and FN007 mutant. Compared with WT nodules, there was no fixation zone (III) in FN007 mutant, but a clearly senescence zone (IV) was emerged. Close-up view of **(B)**, WT and **(E)**, FN007 infected cells. Magnification view of **(C)**, WT and **(F)**, FN007 infected cells, showing degradation of symbiosomes in FN007 infected cells. Scale Bar: **(A, D)**: 100 μm; **(B, E)**: 20 μm; **(C, F)**: 10 μm.

In indeterminate nodules, rhizobia released into nodule cells differentiate before nitrogen fixation begins, and the differentiated rhizobia are morphologically longer than free-state rhizobia and have endoreduplication of their chromosomes (Mergaert et al., 2006). From GFP and *LacZ* stained sections experiments, we found that the FN007 mutant was normal in early nodule development stage including infection thread development and rhizobia release. To further determine the development and differentiation of rhizobia released into nodule cells, 5 μm semithin longitudinal nodule sections were stained with Toluidine blue for microscopic observation. In healthy symbionts, rhizobia undergo intracellular replication and differentiation into rod-like structures evenly distributed around a central vacuole, but rhizobia in infected cells need to differentiate to exert nitrogen-fixing function properly. At 13 dpi, WT nodules (**Figure 5A**) had formed four distinct zones: zone I (zones of meristem, ZI), zone II (infection, ZII), zone II-III (a transition zone between ZII and ZIII, IZ), and zone III (nitrogen fixation, ZIII). Zone II-III was a transition zone from zone II to III, when rhizobia had already entered into infected cells, and with a compartment area of about one-fifth of the total rhizobia area. Infected cells were filled with intact symbiosomes in WT. In this period, the FN007 mutant nodules appeared to have four zones in terms of cell morphology: I, II, IZ, and IV (senescence zone), but did not appear to have the nitrogen-fixing zone (ZIII) as observed in WT nodules, entering directly into the senescence zone. Symbiosomes in most infected cells were disintegrated at the proximal end of the root in the FN007 mutant (**Figure 5D**). In the WT, the senescence zone was formed at 35 dpi in the absence of environmental stress, but in the FN007 mutant, the senescence zone was formed at 13 dpi, which clearly showed an early senescence phenotype. Therefore, it was assumed that symbiotic nitrogen fixation defect occurs in the II-III transition zone, and rhizobia cannot complete the transition after its release into the infected cells in the FN007 nutant. In WT nodules (**Figures 5B, C**), infected cells were morphologically normal, mostly subrounded, with a large number of symbiosomes containing rod-shaped bodies regularly arranged around a large central vacuole. In the FN007 mutant, infected cells (**Figures 5E, F**) were smaller and irregularly shaped compared to that in WT nodules, with less symbiosomes and most were not rod-shaped, resembling the free-living cells.

**Figure 6:**
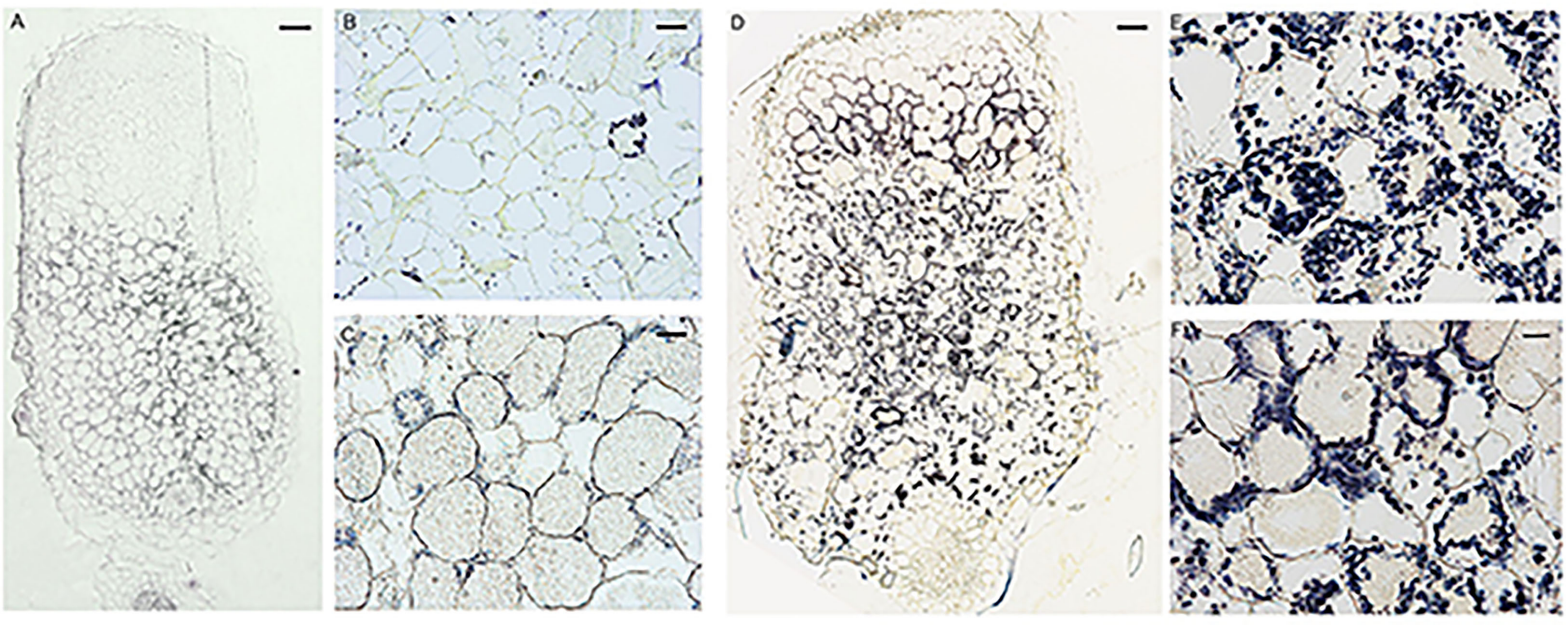
Accumulation of starch granules in FN007 mutant nodules. Potassium iodide staining of starch granules in infected cells in WT nodules **(A–C)** and FN007 mutant nodules **(D–F)**. In WT nodules, starch granules was deposited in large amounts only in the II-III interval zone and in small amounts in the IV zone, while in FN007 mutant nodules, the starch granules deposition was greatly expanded. Scale bar: **(A)**: 100 μm; **(D)**:200 μm; **(B, E)**: 20 μm; **(C, F)**: 10 μm.

To further characterize the symbiotic phenotype of the FN007 mutant, semithin sections of nodules at 13 dpi were stained for starch granules with potassium iodide (KI). The WT nodules had almost no accumulation of starch granules at the distal root end (**Figures 6A, B**), but in the nitrogen fixation zone (**Figure 6C**), there was a small number of starch granules deposition around the cell membrane. Conversely, starch granules in the distal end of the FN007 nodules were evenly distributed and surrounded the cell membrane (**Figure 6D**), where clearly cell contours were observed and the starch granules were similar in size. At the proximal root end of the FN007 mutant nodule (**Figures 6E, F**), the number of deposited amyloplasts was increased and distributed in a disorganized manner. Overall, the number of starch granules in FN007 mutant nodules were greatly exceeded that of the wild type. These results indicated that the FN007 mutant cannot form a functional fixation zone, and thus many metabolic activities can not be carried out in infected cells, which accumulating more energy material (starch grains).

**Figure 7:**
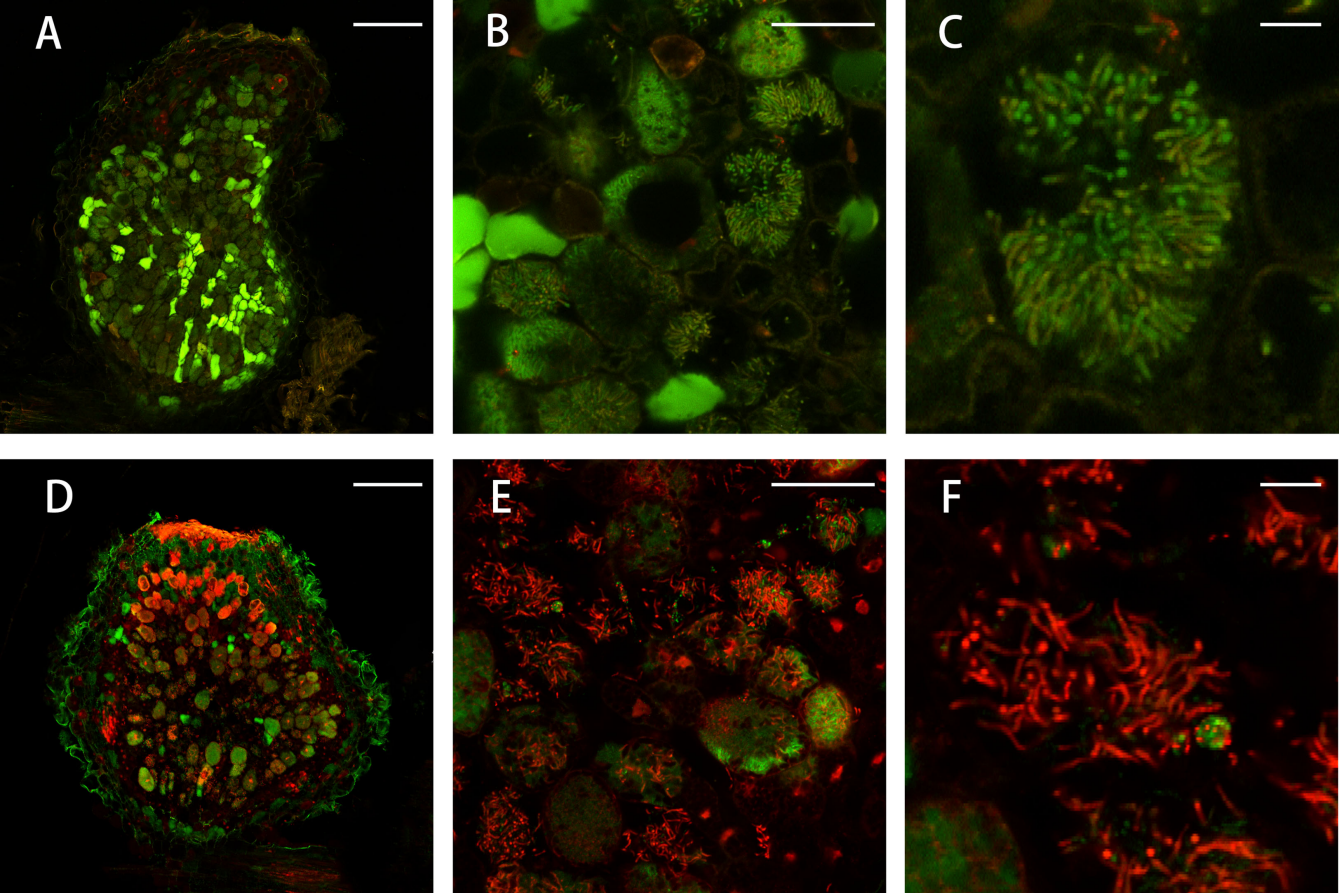
Live/dead staining of nodule cells. Live/dead staining of bacteroids in FN007 mutant compared with WT nodules (13 dpi). **(A–C)**, wild type (WT) and **(D–F)**, FN007 mutant. Live bacteroids have a green signal (GFP) and dying bacteroids have a red signal from propidium iodide (PI). PI can only enter bacteroids with compromised membranes. FN007 mutant nodules fail to sustain differentiated bacteroids. Scale bar: **(A, D)**: 200 μm; **(B, E)**: 50 μm; **(C, F)**: 10 μm.

To confirm whether the FN007 mutant nodules undergo early senescence, we performed live/dead staining experiment, using fluorescence dyes SYTO9, which stains live bacteroids and nucleic acids of plant cells and propidium iodide (PI), which stains dead bacteroids. The FN007 mutant and WT plants were inoculated with *S. medicae* ABS7 strain. A large green fluorescence was observed within the WT nodule (**Figures 7A–C**), and clearly visible symbiosomes were formed within infected cells and regularly arranged around the central vacuole. At 13 dpi, the FN007 mutant nodules were mostly stained red fluorescence compared with the WT (**Figures 7D–F**), and high magnification images showed that infected cells contained both red and green scattered bacteroids, indicating that some bacteroids in the FN007 mutant were dead at this time. These observations suggest that nodule cells of the FN007 mutant exhibited signs of an early senescence phenotype (12 dpi).

### Whole genome sequencing analysis

**Figure 8:**
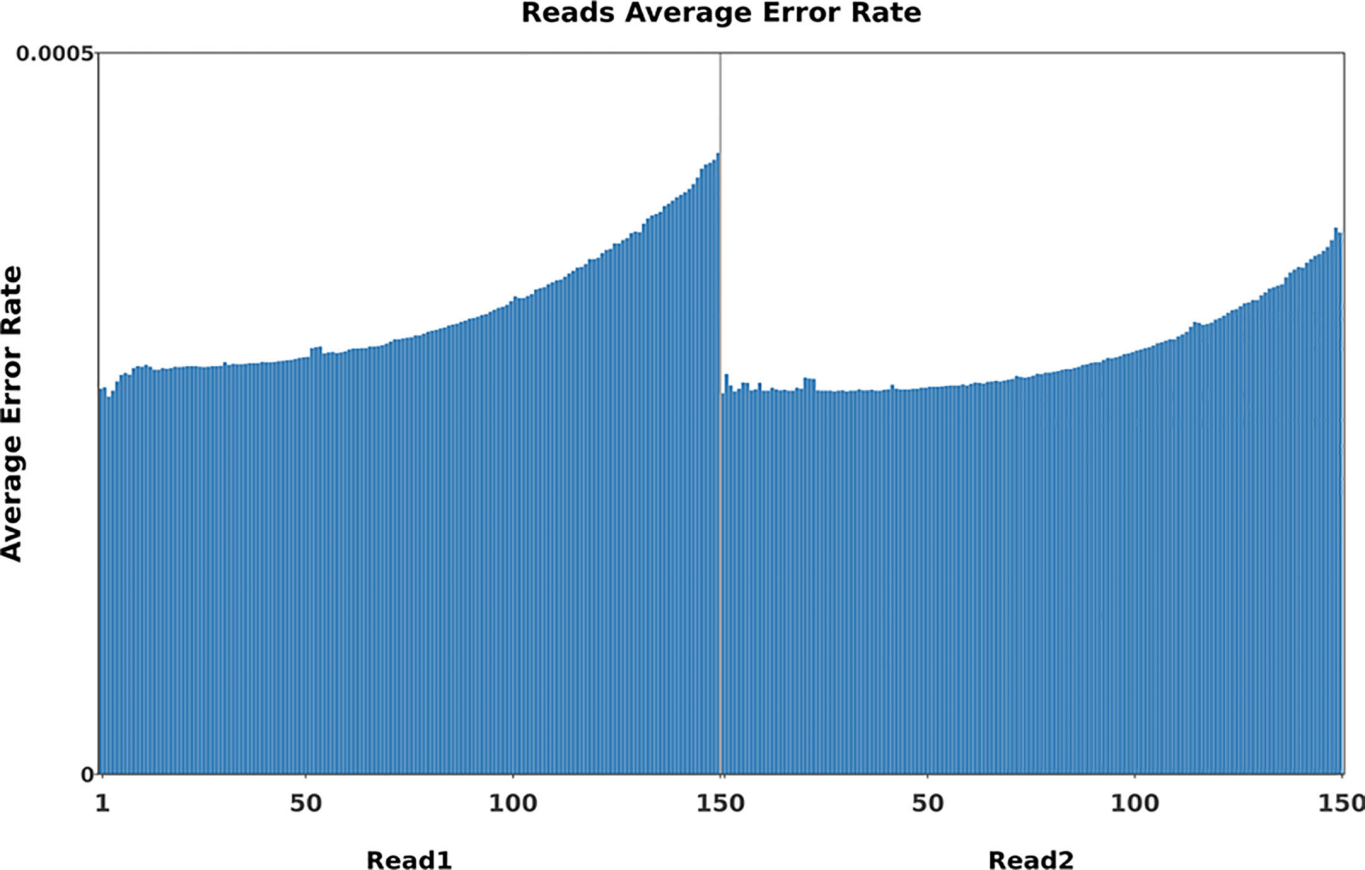
Base error rate distribution graph. Horizontal coordinates represent the base positions on reads, and vertical coordinates represent the average error rate of single bases; the left side shows the base quality distribution of R1 Reads in pair-end sequencing, and the right side shows the base quality distribution of R2 Reads.

To identify mutations in the FN007 mutant, whole genome resequencing was carried out. Raw reads were cleaned after quality control and QC results were obtained (**Table 1**). Normally, for next generation sequencing values, Q20 should be over 95% (at worst, not below 90%), and Q30 is required to be more than 85% (at worst, not below 80%). The sequencing quality of the FN007 mutant and WT was high, with both Q20 values over 97% and both Q30 values over 93% (**Table 1**). The distribution of base error rates for pair-end reads in length range of 1 to 150 bp is shown in **Figure 8**. Regardless of read length, the average base error rate was less than 0.05%, indicating high sequencing quality.

**Table 1:** Statistics of sample sequencing quality control results.

Samples	Raw_ Reads	Clean_ Reads	Clean_Reads_ Percent	Raw_Base	Clean_Base	Clean_Base_ Percent	GC_ Content	>Q20	>Q30
**FN007**	206334558	204918294	99.31%	30950183700 (30.95G)	30378091695 (30.38G)	98.15%	34.79%	97.53%	93.03%
**WT**	164747736	163907076	99.49%	24712160400 (24.71G)	24411975186 (24.41G)	98.79%	34.72%	97.80%	93.83%

Raw_Reads represent Number of original sequencing reads; Clean_Reads represent Number of valid reads after quality control; Clean_Reads_Percent represent Clean_Reads as a proportion of Raw_Reads; Raw_Base represent Amount of raw sequencing data in bp; Clean_Base represent The amount of effective data after quality control in bp; Clean_Base_Percent represent the proportion of Clean_Base to Raw_Base; >Q20 represent the proportion of bases with a quality value greater than Q20 to Clean_Base; >Q30 represent the proportion of bases with a quality value greater than Q30 in Clean_Base.

**Figure 9:**
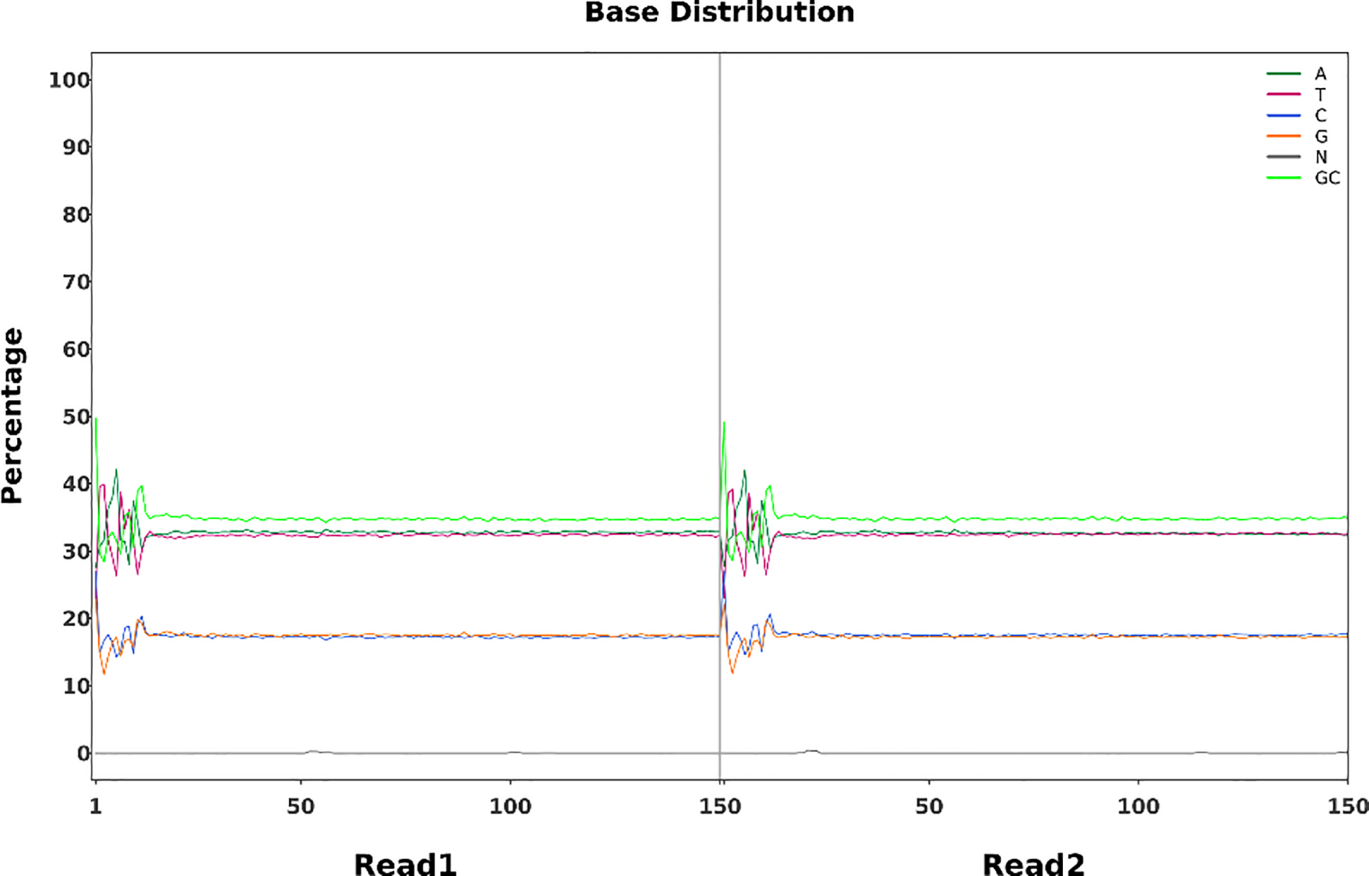
Base percentage distribution. Horizontal coordinates represent base position on the reads, and vertical coordinates represent the proportion of single bases, A,T,C,G,N, and GC, respectively.

Whole-genome sequencing uses non-strand-specific library building. Theoretically, G and C, and A and T bases should be equal in each sequencing cycle, and the whole sequencing process should be stable. The FN007 mutant base distribution sequencing data is shown in **Figure 9**. The start position of the sequence is linked to the primer junction during sequencing, so A, C, G and T will fluctuate at the beginning but stabilize later.

For species with a reference genome, their genomic information or complete transcript information can be downloaded from public databases, and then the sequenced reads can be compared to the reference genome. By quickly comparing each read to the reference genome, information about read position and match quality on the compared genome or other reference sequences can be obtained, and then the genes or transcripts can be annotated and quantified. After comparing the clean reads of the FN007 mutant and WT with the reference genome, the statistical results are shown in **Table 2**. It was suggested that reads from the FN007 mutant is comparable with WT. For the FN007 mutant, more than 189 million pair-end reads (2×150 bp) were produced, which represented a 65.2× sequencing depth. For the WT plant, 151 million reads were produced, representing 52.3× sequencing depth (**Table 2**). These reads were mapped to the latest reference genome of *M. truncatula* (MtrunA17r5.0). The total mean Mapping quality is nearly 50% in both mutant and WT. These result indicated that the WGS data has higher quality of the alignment.

**Table 2:** Statistical table of comparison information between FN007 and WT.

Samples	WT	FN007
Total_reads	163907076	204918294
Dup_reads	10113696	15706947
Dup_percent	6.17%	7.66%
RmDup_reads	153793380	189211347
Mapped_reads	151287765	188990765
Mapped_Rate	98.37%	99.88%
Mean_coverge	52.3507×	65.2098×
Mean_mapping_quality	49.237	49.1109
1×_coverage	99.75%	99.67%
10×_coverage	99.36%	99.36%

Total_Reads represent the number of valid Reads after quality control; Dup_Reads represent the number of PCR duplicate Reads; Dup_percent represent the ratio of PCR duplicate Reads; RmDup_Reads represent the number of Reads after removing PCR duplicates; Mapped_Reads represent the number of Reads on the reference genome; Mapped_Rate represent the ratio between Mapped_Reads and RmDup_Reads; mean_coverage represent average coverage depth, calculated as the total number of bases paired to the reference genome/reference genome length; mean_mapping_quality represent the higher the value, the better the quality of the alignment; 1×_coverage represent the percentage of loci covered by at least 1 Reads in the reference genome to the whole length of the genome, i.e. the genome coverage; 10×_coverage represent the number of loci covered by at least 10 Reads in the reference genome as a percentage of the total genome length.

**Figure 10:**
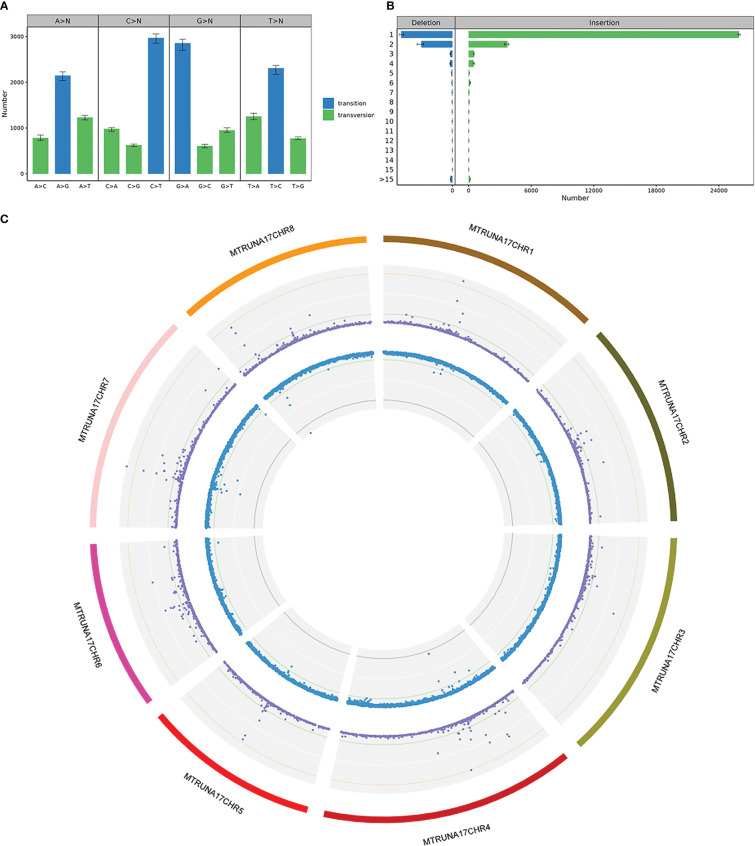
SNP & InDel Variant Detection in FN007 mutant **(A)** SNP substitution type statistics in FN007 mutant. Horizontal coordinates represent SNP substitution type, vertical coordinates are the mean, maximum and minimum values for each sample, and colors indicate whether SNP substitution type is a transition or a transversion. **(B)** InDel substitution type length statistics in FN007 mutant. Horizontal coordinates show the mean, maximum and minimum values of the InDel numbers, vertical coordinates show substitution base lengths, and colors indicate whether the substitution type is insertion or deletion. **(C)** Circos visualization of identified deletions in FN007 mutant. SNP and InDel distributions on individual chromosomes, where the first circle represents the FN007 chromosome, the purple dots in the second circle represent SNP density, and blue dots in the third circle represent InDel density.

SNP and InDel detection were performed using the GATK4 software Haplotypecaller module, based on the comparison of samples with the reference genome. QD is the ratio of variant quality divided by the depth of coverage, which is actually variant quality per unit depth, and most false positive variants have a QD of less than 2. Compared to transversion, transition is the most common mutation type, with T to C and A to G being the most common substitution types (**Figure 10A**). Of all the InDels obtained, deletions less than 4 bp accounted for 85.28% and insertions less than 4 bp accounted for 94.53% (**Figure 10B**). All the mutations detected were integrated into a Circos plot to visualize the genomic data (**Figure 10C**).

**Figure 11:**
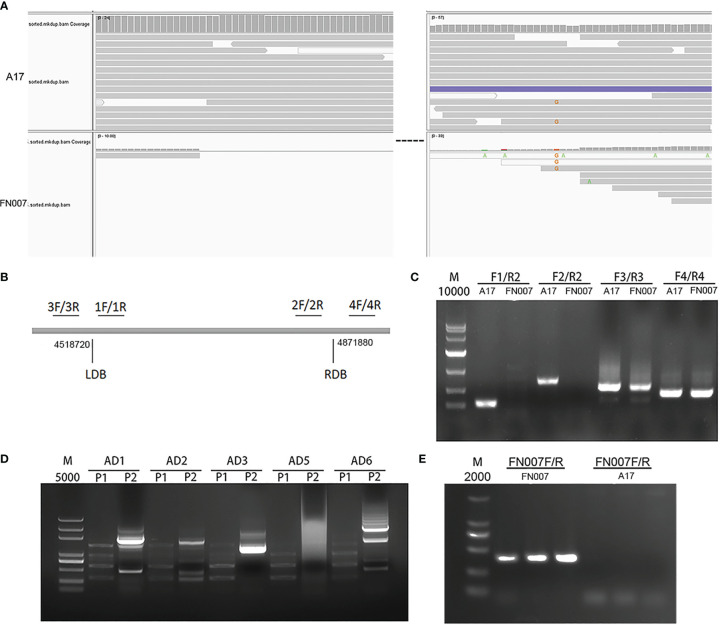
Identification of copy number variation (CNV) in FN007 mutant. **(A)** Visualization of the causal deletion using IGV. The reads alignment for WT and FN007 mutant. The sequencing showed a large deletion at the corresponding position in FN007 mutant; **(B)** Coordinate positions between four primers are designed for the identification of deletion border in FN007 mutant; **(C)** PCR detection for deletion junction through the flanking sequencing primers in FN007 mutant; **(D)** Tail-PCR was carried out, and the corresponding band were extracted for sequencing; **(E)** Deletion border confirmed by PCR in FN007 mutant. A pair of primer (FN007F/FN007R) was designed at deletion junction and amplified in FN007 mutant and WT. The results showed that FN007F/R could amplify the target band in FN007 mutant but not in WT.

The FN007 mutant resequencing data revealed that there was a copy number variation (CNV) in large segment of chromosome 3. CNVs may be biologically important for species-specific genome composition, species evolution and phylogeny, as well as for the expression and regulation of genes in specific genome regions. We used IGV software to visualize detection CNVs in the FN007 mutant, and found a possible large deletion on chromosome 3 from location 4,518,720 to 4,871,880 (**Figure 11A**). To determine the specific interval of the mutant deletion, four pairs of primer (**Table S1**) were first designed on both sides of each proposed boundary provided by the IGV data, and these primers were amplified by PCR in the FN007 mutant and WT (**Figure 11B**). The results showed that primers 3 and 4 could amplify the target bands in the FN007 mutant, but in contrast, primers 1 and 2 could not (**Figure 11C**). This suggests that the IGV data matches the actual deletion junction in the FN007 mutant. To identify the exactly deletion site, TAIL PCR was performed to identify the deletion regions in the FN007 mutant (**Figure 11D**). Results showed that the deletion border in the FN007 mutant was located at position 4,519,828-4,872,290 on chromosome 3. Based on the TAIL PCR results, we designed left and right border primers and verified the results, conformed that there was a deletion region in the FN007 mutant compared with WT (**Figure 11E**).

### Genetic linkage analysis

Whole genome sequencing analysis revealed that the FN007 mutant has a large deletion of approximately 348 kb on chromosome 3. To characterize the nature of the FN007 mutation, we generated F2 populations from cross-pollination between the FN007 mutant and its WT parent (Jemalong A17).

**Figure 12:**
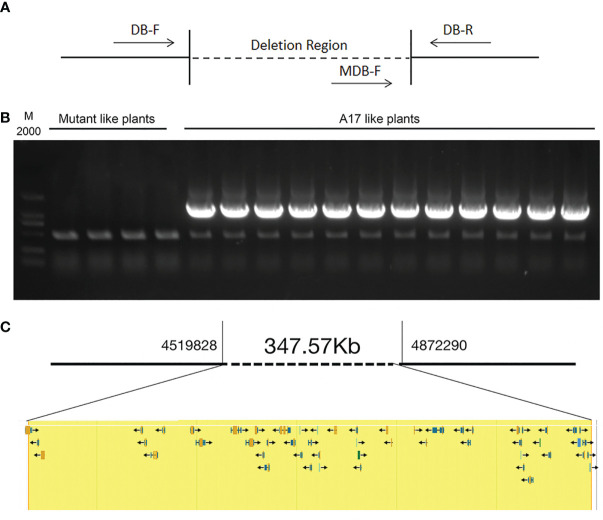
Genetic linkage analysis of FN007 mutant A genetic linkage analysis of FN007 F2 segregating population. **(A)** PCR analysis was carried out using primers spanning the deletion border in FN007 F2 population; **(B)** Shown are PCR amplification of deletion border in F2 mutant pools (mutant like plants, 10 plants per pool) and in wild type-like pools (WT like plants, 10 plants per pool). M, DL2000, molecular weight markers. There were no specific bands in F2 mutant pool by MDB-F and DB-R primer sets, while in WT like pools, all of pools could amplify the correct band. These results indicate that the deletion region has linkage with FN007 mutant phenotype; **(C)** A putative 348 kb deletion segment in FN007 mutant.

We analyzed 163 individual F2 plants and observed a ratio of 3.075:1 segregation of WT and mutant nodulation phenotypes in F2 populations, indicating a single recessive locus for the FN007 mutant phenotype. Next, we performed a linkage analysis, using pools of DNA samples isolated from individual F2 plants. The 123 plants with the WT phenotype were divided into 12 groups and 40 plants with the mutant phenotype were divided into 4 groups (the DNA from each group was pooled together). The deletion regions were identified by PCR using the corresponding primers (**Figure 12A**). The results showed that all the mutants plant could amplify the deletion border by MDB-F and MDB-R primer (data not shown), but could not get the corresponding band by the MDB-F and DB-R primer, while the WT plants can amplify the correct bands in the deletion region (**Figure 12B**). These results indicated that the fix^-^ phenotype of the FN007 mutant was tightly linked to the deleted region on chromosome 3.

Within the deletion region on chromosome 3, there are 53 annotated genes base on *M. truncatula* A17 r5.0 genome (**Figure 12C**; **Table 3**). This, together with the results of the genetic linkage analysis, suggests that these genes are candidates for the cause of the phenotype of the FN007 mutant. Though we have successfully identified the deletion region in the FN007 mutant, it will take more effort to pinpoint the causal gene. To identify which is the target gene, RNA-seq technology would be used to narrow down the gene list in the deletion region. Finally, genetic complementation assay should be carried out, using *Agrobacterium rhizogenes*-mediated root transformation.

**Table 3:** Gene list in deletion region of FN007 mutant.

No.	Start	End	Gene name	Annotation
1	4517833	4521967	MtrunA17Chr3g0082791	Putative P-loop containing nucleoside triphosphate hydrolase%2C leucine-rich repeat domain%2C L
2	4525308	4526227	MtrunA17Chr3g0082801	hypothetical protein
3	4527738	4530138	MtrunA17Chr3g0082811	Putative P-loop containing nucleoside triphosphate hydrolase%2C leucine-rich repeat domain%2C L
4	4590083	4591230	MtrunA17Chr3g0082821	hypothetical protein
5	4592379	4593528	MtrunA17Chr3g0082831	hypothetical protein
6	4596357	4600625	MtrunA17Chr3g0082841	Putative tetratricopeptide-like helical domain%2C thioredoxin-like protein
7	4603651	4605160	MtrunA17Chr3g0082851	hypothetical protein
8	4621143	4623476	MtrunA17Chr3g0082861	Putative response regulator and transcription factor RR-A-type family
9	4624537	4630582	MtrunA17Chr3g0082871	Putative P-loop containing nucleoside triphosphate hydrolase%2C leucine-rich repeat domain%2C L
10	4646449	4653195	MtrunA17Chr3g0082881	Putative P-loop containing nucleoside triphosphate hydrolase%2C leucine-rich repeat domain%2C L
11	4655741	4660843	MtrunA17Chr3g0082891	Putative P-loop containing nucleoside triphosphate hydrolase%2C leucine-rich repeat domain%2C L
12	4661521	4663557	MtrunA17Chr3g0082901	Putative transcription factor interactor and regulator CCHC(Zn) family
13	4666281	4667366	MtrunA17Chr3g0082911	hypothetical protein
14	4667573	4670400	MtrunA17Chr3g0082921	Early nodulin-10
15	4669003	4669760	MtrunA17Chr3g0082931	hypothetical protein
16	4676905	4683938	MtrunA17Chr3g0082941	Early nodulin-12A
17	4684026	4686560	MtrunA17Chr3g0082951	Putative Gnk2-like domain-containing protein
18	4689411	4689819	MtrunA17Chr3g1009661	hypothetical protein
19	4689934	4691347	MtrunA17Chr3g0082961	hypothetical protein
20	4694891	4697009	MtrunA17Chr3g0082971	hypothetical protein
21	4695914	4696898	MtrunA17Chr3g0082981	Early nodulin-12A
22	4700523	4700914	MtrunA17Chr3g1009664	hypothetical protein
23	4701367	4702332	MtrunA17Chr3g0082991	hypothetical protein
24	4701570	4702308	MtrunA17Chr3g1009666	hypothetical protein
25	4711121	4712887	MtrunA17Chr3g0083001	Putative Serpin family protein
26	4724803	4725523	MtrunA17Chr3g0083011	Putative Late nodulin
27	4724972	4725480	MtrunA17Chr3g1009670	hypothetical protein
28	4725879	4727343	MtrunA17Chr3g0083021	hypothetical protein
29	4727375	4727763	MtrunA17Chr3g0083031	hypothetical protein
30	4743584	4744548	MtrunA17Chr3g0083041	Putative Serpin family protein
31	4746749	4747099	MtrunA17Chr3g0083051	hypothetical protein
32	4761026	4761329	MtrunA17Chr3g0083061	hypothetical protein
33	4768494	4768931	MtrunA17Chr3g0083071	hypothetical protein
34	4772616	4779909	MtrunA17Chr3g0083081	Putative Late nodulin
35	4790225	4791820	MtrunA17Chr3g0083091	Putative Late nodulin
36	4794477	4796323	MtrunA17Chr3g0083101	hypothetical protein
37	4797383	4797805	MtrunA17Chr3g0083111	Putative Late nodulin
38	4825327	4826727	MtrunA17Chr3g0083121	Putative transcription factor interactor and regulator CCHC(Zn) family
39	4826893	4828195	MtrunA17Chr3g0083131	Putative Late nodulin
40	4827653	4828070	MtrunA17Chr3g1009692	hypothetical protein
41	4828580	4829067	MtrunA17Chr3g0083141	hypothetical protein
42	4829203	4829717	MtrunA17Chr3g0083151	hypothetical protein
43	4832527	4835586	MtrunA17Chr3g0083161	hypothetical protein
44	4839696	4840116	MtrunA17Chr3g0083171	hypothetical protein
45	4843168	4844486	MtrunA17Chr3g0083181	hypothetical protein
46	4845159	4846684	MtrunA17Chr3g0083191	hypothetical protein
47	4860530	4862920	MtrunA17Chr3g0083201	hypothetical protein
48	4863407	4865364	MtrunA17Chr3g0083211	hypothetical protein
49	4864538	4865110	MtrunA17Chr3g0083221	hypothetical protein
50	4866497	4868953	MtrunA17Chr3g0083231	hypothetical protein
51	4866613	4867679	MtrunA17Chr3g0083241	hypothetical protein
52	4869407	4870204	MtrunA17Chr3g0083251	hypothetical protein
53	4870951	4871406	MtrunA17Chr3g0083261	hypothetical protein

## Discussion

Leguminous plants are able to establish symbiotic relationships with the soil bacteria rhizobia and as a result, develop nodules on their roots, in which biological nitrogen fixation take place. The development of nodules on roots of host plants is triggered by the perception of Nod factors by receptor-like kinase complexes of compatible host plants (Oldroyd et al., 2011; Zipfel and Oldroyd, 2017). Within cytoplasm of plant cells, rhizobia are enclosed by the plant-derived membrane, symbiosome membrane and differentiate into nitrogen fixing bacteroids, and symbiotic nitrogen fixation occurs. Later on, nodule cells senesce and nitrogen fixation stop (Puppo et al., 2005). In *M. truncatula*, nodules formed on roots are indeterminate. They are cylindrical and contain a series of development zones of different cell types. In mature nodules, there is a senescence zone, where the host cells and bacteroids degenerate. For most leguminous plants, significant nodule senescence is observed during pod filling, when nutrient reallocation and remobilization occur to ensure yield and seed quality. Therefore, increasing active nitrogen fixation in nodules by delaying their senescence might be a powerful strategy to improve nitrogen-use efficiency during seed development (Yu et al., 2023). However, the key factors that orchestrate nodule senescence have not been identified (Wang et al., 2022).

In this study, we isolated and characterized a new nodulation mutant from *M. truncatula*, FN007. The FN007 mutant was isolated as being defective in symbiotic nitrogen fixation from a *M. truncatula* deletion mutant collection generated by fast neutron bombardment. Phenotypic characterization showed that the FN007 mutant was defective in later stage of nodule development. In contrast to WT plants, which develop rod shape nodules, the FN007 mutant plants develop round, spherical nodules. A possible explanation for this could be that the deletion of the target gene affects the expression of the leghemoglobin gene in FN007 mutant (Jiang et al., 2021), resulting in white root nodules at all stages of development, and that the expression of genes involved in root nodule development is restricted (Li et al., 2022).

Microscopic observations of the FN007 mutant nodules showed the presence of the meristem zone, infection zone, transition zone, and senescence zone without a functional fixation zone. The symbiosomes and infected cells were degraded starting about 12 dpi. The number of nodules was much higher and the distribution of nodules was scattered in the FN007 mutant than WT plants after inoculation with rhizobia. These observations suggest that nodules developed on the FN007 mutant roots undergo early senescence.

Mutagenesis is a powerful tool for gene functional studies. Fast neutron, a high energy ionization mutagen, has been used in generating mutants in many plant species. A large portion of mutations derived from FN mutagenesis are due to DNA deletions that range in size from a few base pairs to mega base pairs. Many phenotype-associated genes have been successfully identified and characterized. Previously, molecular cloning of the underlying genes from FN mutants relied on a map-based approach, which is time -consuming and limits the number of mutants to be characterized at the molecular level. Recently, several complimentary approaches including genome tiling array-based comparative genomic hybridization (CGH) for DNA copy number variation detection, has been employed to facilitate the characterization of deletion mutants in diverse organisms including animals and plants. These approaches provided new solutions in characterizing with FN mutants. However, low resolution and low accuracy, especially for small deletions limited these methods to apply in identifying mutations for FN mutants.

With the rapid development of next generation sequencing (NGS) technologies, whole genome sequencing (WGS) has become more affordable, providing a new means of identifying causal deletions in FN mutants (Sun et al., 2018). WGS for FN mutants not only increases the sensitivity in detecting mutations on chromosomes, but also improves the reliability and resolution in identifying deletions (Du et al., 2021). In this study, we used it to analyze symbiotic nitrogen fixation deficiency mutant FN007 in *M. truncatula* and identified the deletion locus that was tightly linked with this phenotype. The causal deletion in the FN007 mutant was confirmed by Tail-PCR amplification, sequencing and genetic linkage analyses.

WGS can be used for large and small deletion identification in many species with reference genome sequences. WGS uses high coverage short reads to examine the large deletions or indel between the reference genome and short reads. In this study, we performed a 30×sequencing depth for mutation detection. Given sufficient sequencing depth, WGS could theoretically detect all potential deletions or indel caused by different mutagenesis. WGS provided a powerful platform to rapidly identify the candidate deletions for FN mutants.

In this study, the FN007 mutant showed a clearly different phenotype from WT under nitrogen-limited and symbiotic condition, so further use of the hairy root transformation technique to rescue the phenotype of the mutant and find the target gene is very important to elucidate the mechanism of symbiotic nitrogen fixation in leguminous plants. This work will provide a reference for most researchers to investigate similar mutant and explore the molecular mechanism of symbiotic nitrogen fixation in leguminous plants.

## Conclusions

*M. truncatula* has been selected as model species for legume-rhizobial symbiosis and nodule development studies. Here, we describe the detail procedures that are being used to characterize SNF mutants in *M. truncatula*, which provide a reliable solutions to utilize FN mutants for SNF research. Our results demonstrate that the methods used here is feasible in screening and identifying the mutation sites for FN mutants in *M. truncatula.*

## Data availability statement

The data presented in the study are deposited in the NCBI Sequence Read Archive (SRA) repository, accession number is PRJNA983295. The release date is: 2023-06-14. The SRA records will be accessible with the following link: https://www.ncbi.nlm.nih.gov/sra/PRJNA983295.

## Author contributions

YC, YS and YM conceived the original research plans. YM, YS, DL, YP and MK performed all the phenotype analysis. YM, YS, DL, WT, RZ, SH, WS and YL performed all bioinformatics analysis. WH and DW performed most of the experiments. YC, YS and YM drafted, wrote, and edited the manuscript. All authors contributed to the article and approved the submitted version.
