# Supplementary material for: Biological and genomic analysis of a symbiotic nitrogen fixation defective mutant in Medicago truncatula
Source: Front Plant Sci. 2023 Jun 28;14:1209664. doi: 10.3389/fpls.2023.1209664 (PMC10345209; doi:10.3389/fpls.2023.1209664)
Supplement: Supplementary file 1 [file Table_1.docx]

**Table S1. Primer sequences and their Tm values**

|  | Sequence (5'-3') | Tm |
| --- | --- | --- |
| 1F | CAGACTCAAGATTGTTTGTCAG | 53℃ |
| 1R | CTTTAGAACTAGAAGCTTCGG | 52℃ |
| 2F | CAACTCTATTGGAGCTGAAAGC | 55℃ |
| 2R | GACCTTTGTTTCTCTTTTGCT | 53℃ |
| 3F | GAGTCTGAATGCGCATGAAT | 56℃ |
| 3R | GCTTTCAGTTCCAATAGAGTT | 53℃ |
| 4F | GGCTGGTAGATGGTCTGAA | 54℃ |
| 4R | CCCAAGTCCATCTGCTGTA | 54℃ |
| LDB-P1 | GAGTCTGAATGCGCATGAATCCTCTAAGAA | 61℃ |
| LDB-P2 | GCAAGAGGGGTGGTTATAAG | 55℃ |
| RDB-P1 | TATAACATGAAGTATTCACCGCTTCTGCCC | 61℃ |
| RDB-P2 | CCACCATGATCACTTCTGAC | 56℃ |
| AD1 | NGTCGASWGANAWGAA |  |
| AD2 | TGWGNAGSANCASAGA |  |
| AD3 | AGWGNAGWANCAWAGG |  |
| AD5 | TCSTICGNACITWGGA |  |
| AD6 | WGTGNAGWANCANAGA |  |

Note: (1) Four pairs of primer (primers 1-4) were designed on both sides of each proposed boundary provided by the IGV data for the purpose of determining the specific interval of the mutant DNA deletion. (2) To determine the specific deletion boundary of the mutant FN007, primer P1 and P2 were designed at the ends of the proposed left and right boundaries, respectively. The PCR product was sequenced and compared with the genome of *M. truncatula* wild type A17 to confirm the deletion borders in the FN007 mutant.
